# Cognitive Strategies and Natural Environments Interact in Influencing Executive Function

**DOI:** 10.3389/fpsyg.2018.01248

**Published:** 2018-07-23

**Authors:** Stefan C. Bourrier, Marc G. Berman, James T. Enns

**Affiliations:** ^1^Department of Psychology, University of British Columbia, Vancouver, BC, Canada; ^2^Department of Psychology, University of Chicago, Chicago, IL, United States; ^3^School of Psychological Science, University of Western Australia, Perth, WA, Australia

**Keywords:** attention restoration theory, executive functioning, natural environments, cognitive strategies, visual attention, additive factors logic

## Abstract

Exposure to natural environments and the adoption of specific cognitive strategies are each claimed to have a direct influence on executive mental functioning. Here we manipulate both factors to help determine whether they draw on common cognitive resources. Three experiments investigated links between environmental effects (nature vs. urban video tours) and strategic effects (active vs. passive instructional approaches to the task). Each experiment used a pretest-posttest design and assessed executive mental functioning using a backward digit span task and Raven's progressive matrices. Experiment 1 manipulated participants' cognitive strategy through explicit instructions in order to establish a link between cognitive strategy and executive mental functioning. Experiment 2 used a pair of 10-min video tours (urban, nature) to examine the relationship between environmental exposure and executive mental function on the same tasks, replicating previous findings with the backward digit span task and extended them to a new task (i.e., Raven's progressive matrices). In Experiment 3, these two manipulations were combined to explore the relations between them. The results showed that the nature video tour attenuated the influence of task instructions relative to the urban video tour. An interaction between environmental video exposure and cognitive strategy was found, in that effects of cognitive strategy on executive function were smaller in the nature video condition than in the urban video condition. This suggests that brief exposure to nature had a direct positive influence on executive mental functioning.

## Introduction

The past decade has seen considerable interest in the relations between the natural environment and human cognition (Kaplan and Berman, 2010; Bratman et al., 2015; Dadvand et al., 2015; Kabisch et al., 2015). Some of the research is driven by concerns of rapid environmental change (Gifford, 2011), but other research focuses on the roots of people's interest in a closer connection to nature. This connection is seen in the growing interest in maintaining gardens (Clatworthy et al., 2013), bringing plant life indoors (Bringslimark et al., 2009), and seeking time to enjoy green spaces (Kaplan and Kaplan, 1989). A recent literature review summarizes many health effects of experiencing natural environments (Hartig et al., 2014), including that spending time in nature reduces stress (Hartig et al., 2014), reduces depressive symptoms (Berman et al., 2012), contributes to positive general health outcomes (Mitchell and Popham, 2008; Kardan et al., 2015), and increases work productivity and enjoyment (Raanaas et al., 2011). Of particular interest to cognitive researchers are claims concerning the benefits to cognition (e.g., attention, working memory, executive functions) that can be delivered through the experience of nature.

As in many fields of research, theoretical and mechanistic understanding of the main findings often lags behind the evidence for positive outcomes. This leaves many unanswered questions. Here we focus on whether the restorative effects of natural environments occur through a direct influence on the operations of central executive functioning, as originally claimed by Kaplan (1995) and reiterated by Kaplan and Berman (2010). The primary support for this claim comes from many studies (Bodin and Hartig, 2003; Cimprich and Ronis, 2003; Stark, 2003; Berman et al., 2008, 2012; Perkins et al., 2011; Emfield and Neider, 2014; Gamble et al., 2014; Lin et al., 2014; Bratman et al., 2015; Rogerson and Barton, 2015; Gidlow et al., 2016; Li and Sullivan, 2016; Triguero-Mas et al., 2017) reporting a link between executive functioning and exposure to natural vs. built environments (via walks, videos, and still photos). Many of these studies report differential effects on a particular cognitive task, the backward digit span task, which is a popular measure in the literature on executive functioning.

### Interactions between restorative environments and cognitive strategies

We view restorative environments as a broad class of environments that have the potential to improve or restore human psychological functioning. Of these, the most relevant for this study is the natural environment. Several theories have proposed mechanisms for why and how interactions with some environments offer psychological improvements, with attention restoration theory (ART) hypothesizing that interactions with nature restore or improve functioning of goal-directed attention mechanisms. But there are other theories worth noting as well. The biophilia hypothesis (Kellert and Wilson, 1995) proposes that interactions with nature satisfy inherent and innate human drives to connect with nature. These ideas are similar to theories claiming that the benefits of engaging with nature are mediated through a sense of nature-connectedness (Mayer et al., 2009), and to theories appealing to an adaptation to natural environments through our evolutionary history (Kaplan, 1995), and to theories appealing to processing fluency (Joye and Van den Berg, 2011).

However, there are other theories proposing that the key process in restoration involves stress reduction via a feedback loop connecting our cognitive systems with affective arousal (Ulrich, 1983). In particular, the stress reduction theory (SRT) posits that interacting with nature promotes relaxation and physiological recovery from stress (Ulrich, 1983; Ulrich et al., 1991). In this view, individuals who experience high states of stress or anxiety are those who also stand to show the most pronounced effect of natural environmental exposure. In fact, participants diagnosed with clinical depression and high anxiety comorbidities showed larger benefits after interactions with nature than participants without those diagnoses (Berman et al., 2012). These arguments are in line with other research reporting affective benefits of built spaces (Ouellette et al., 2005; Karmanov and Hamel, 2008), though it is still unclear how potential affective and cognitive benefits are related.

The present study was motivated by ART and the cognitive benefits linked to interacting with nature. A central tenet of ART is that the central control processes of attention are susceptible to fatigue after use, while involuntary attention selection mechanisms are less susceptible to fatigue (Kaplan, 1995; Berman et al., 2008; Kaplan and Berman, 2010). As such, environments that place few demands on directed attention, while simultaneously having interesting stimulation to capture involuntary attention, create situations that can help to restore or replenish the limited mental resources of directed attention (Kaplan, 1995; Kaplan and Berman, 2010). From the perspective of ART, natural environments tend to make fewer harsh demands of attention for active cognitive control than urban environments. Indeed, natural environments are said to be “softly fascinating,” (Kaplan, 1992, p. 139) which refers to the capture of attention by exogenous mechanisms that do not require effortful control (i.e., one can look at a waterfall, but still attend to other things; Kaplan and Berman, 2010). This is in contrast to the built environment, which tends to have multiple salient cues that capture attention simultaneously, and must therefore be processed with effort (e.g., car horns, sirens, billboards).

One way an environment may be softly fascinating concerns the predictable relations that can be found among its elements. For example, in a forest environment green undergrowth is topped by a sparser set of larger trees that open into a canopy through which the sky is occasionally visible. The shapes, patterns, and textures tend to be related to one another through self-similarity and mutual organic necessity (Mandelbrot, 1977), an argument corroborated by the fact that natural environments tend to have higher fractal dimensionality than urban ones (Hagerhall et al., 2015). Urban environments tend to have many salient cues (singular events and objects amid surroundings that are less predictable from one another) calling for the attention of the participant in a way that requires effortful selection in order to pay attention to one cue and/or to ignore the irrelevant cues (Hagerhall et al., 2004). Research on eye-tracking is consistent with this view, showing that urban images rated low on fascination increased exploratory eye-movements relative to image of nature (Berto et al., 2008). It is as though more effort is required in an urban setting to engage volitional control processes. Berto's (2014) extensive review of the literature emphasizes: (1) that urban environments place greater demands on sustained attention than natural environments, (2) that sustained attention is effortful and leads to mental fatigue, an argument that is line with Kaplan and Berman (2010); and (3) that it is unclear at present whether active or passive cognitive engagement with natural environments leads to greater restorative benefits.

At the same time, we note that ART does not claim that the environment must be natural *per se* to be restorative (Kaplan and Berman, 2010). The environment just needs to meet the criteria of not taxing top-down directed attention, while simultaneously having softly fascinating stimulation to capture involuntary attention (Kaplan and Berman, 2010). This leaves open the possibility that walking through a museum, or interacting with other built spaces that have beautiful architecture with limited distractions, could be restorative (Kaplan et al., 1993; Ouellette et al., 2005).

### Executive function and cognitive strategy

Executive functioning is an umbrella term that has conventionally been used to refer to cognitive operations that require conscious effort and control, including switching between one task and another, retrieving task relevant episodes from long term memory (Logie et al., 2004; Baddeley, 2007), performing operations in working memory (Baddeley and Hitch, 2010) and staying on task in the face of potentially distracting events or thoughts (Engle et al., 1999; Miyake et al., 2000). Two of the most popular measures of executive functioning are the backward span digit task (Lehto, 1996; Baddeley and Hitch, 2010) and Raven's progressive matrices (Raven, 1989; Carpenter et al., 1990).

The explicit adoption of control strategies has been shown to influence cognitive performance, though these effects are thought to be under the participant's direct control rather than being imposed softly by the environment. For example, adopting so-called *passive* vs. *active* control strategies has been shown to influence tasks of object categorization (Jacoby and Brooks, 1984; Whittlesea et al., 1994) and visual search (Smilek et al., 2006; Watson et al., 2010). In both of these literatures, participants are instructed to take either a *passive* cognitive approach (e.g., “let the answer just pop into your mind”) or an *active* one (e.g., “actively direct your attention to specific features and objects”). In object categorization, a passive strategy is linked to results that are based on family resemblance and global characteristics of stimuli, whereas an active strategy is linked to feature- and rule-based groupings of stimuli. Whether these results are beneficial or harmful to overall performance depends on the task constructed by the experimenter (i.e., whether there is an objective correct answer that is holistic or feature-based). This research hints that an active strategy, relative to a passive strategy, leads to greater involvement of central cognitive control. This is consistent with Smilek et al. (2006), who claimed that executive function involvement interferes with efficient visual search, and with Jacoby and Brooks (1984), who claimed that greater executive control is required for rule-based categorization than for similarity based categorization.

### Additive factors logic

Although attention restoration theory claims that exposure to nature restores central executive function, this may not be the only mechanism responsible for improved performance on tasks such as digit span. For example, nature could have an influence through an overall change in task motivation, or on the amount of effort participants are willing to apply to the task, or because recent experiences bias the way that sensory information is registered for further central processing, or because those experiences bias the response mode of the participant. Importantly, these other possible factors do not have direct links in most theoretical formulations to the operation of central executive functioning (Engle et al., 1999; Miyake et al., 2000; Logie et al., 2004; Baddeley, 2007). In these theories, central executive functioning has its inputs from sensory processes and its outputs to response selection mechanisms.

**Figure 1:**
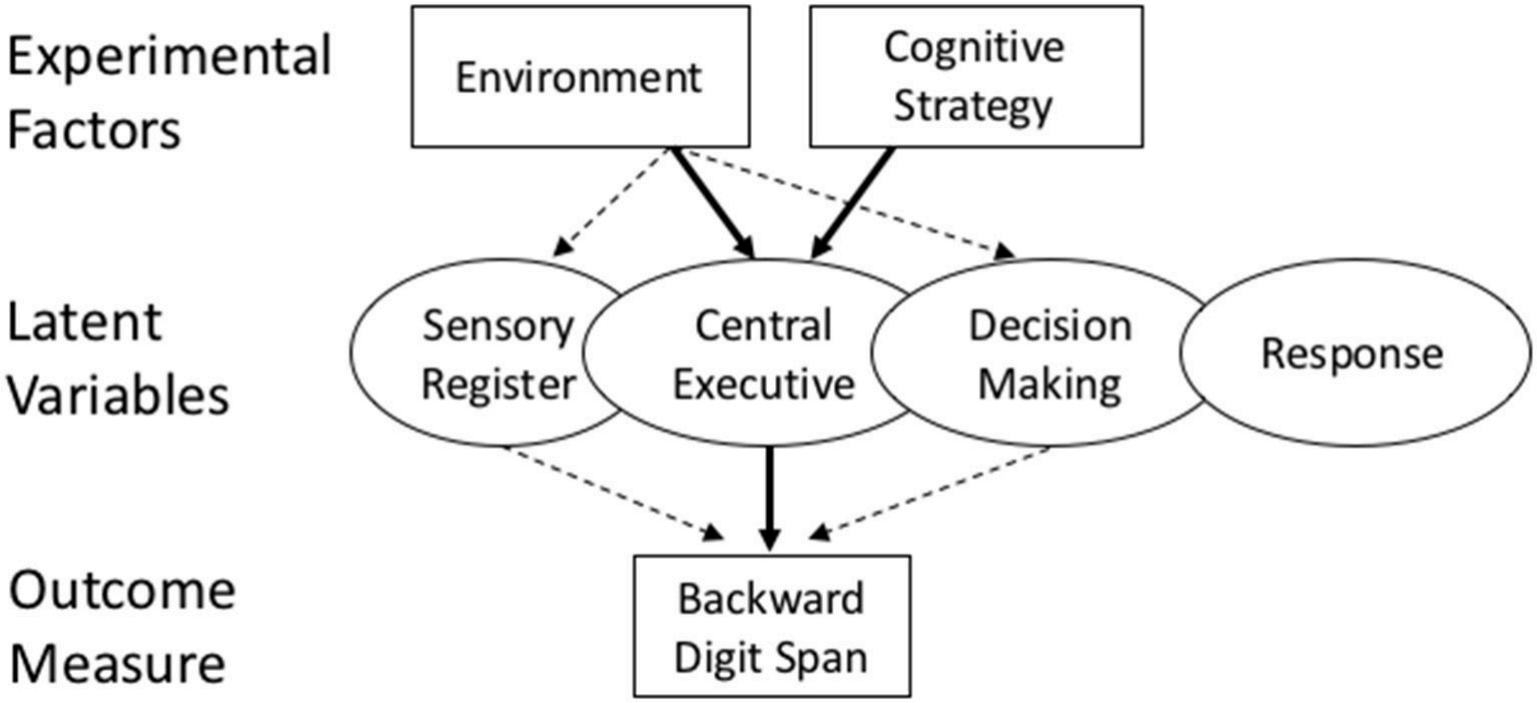
A schematic of the experimental design and hypothesis being tested in this study. Attention restoration theory claims that natural environments (an experimental factor) influence backward digit span tasks (an outcome) through their effect on central executive functioning (a latent variable). A strong test of this hypothesis is given by simultaneously varying another experimental factor (cognitive strategy) which is also shown to influence executive functioning. If the environment has an influence on other latent variables (e.g., sensory register, decision making) then additive results are possible, because each experimental factor is influencing a different latent variable. If both experimental factors influence the same latent variable, then interactive results are predicted.

The central aim of this study is to use additive factors logic, as illustrated in Figure 1, to test the hypothesis that exposure to nature has its influence on a digit span task through a specific latent variable, the available capacity to perform mental operations in working memory. This hypothesis has been supported in past studies documenting that manipuations of the environment have consequences for digit span memory (Stenefors et al., under review). But these data do not rule out the possibility that other factors that covary with exposure to nature play a role as well. These potential covariates, such as differences in sensory registration, decision making, or response processes, could exert their influence in advance, or even following, the processes of executive functioning, and could therefore contribute to the changes in outcome without necessarily influencing executive functioning *per se*.

Additive factors logic asserts that stronger confirmatory evidence can be obtained—for the claim that a latent construct such as executive functioning is causally involved in the digit span outcome—by manipulating executive functioning by an independent means. In the present study, we have chosen to do so by varying another experimental factor that is well established to influence executive functioning (instructions on strategy use) and then seeing whether the environment and cognitive strategy have interactive influences on digit span memory. This is the heart of additive factors logic (Sternberg, 1969, 1998). If two experimental factors don't interact (they influence an outcome independently), then it leaves open the possibility that the environment does not influence digit span through the central executive. One of those other latent variables may still be the leading cause of the nature-digit span relationship (as shown by the dashed arrows in Figure 1). However, if the environment and cognitive strategy are synergistic in influencing digit span (there is an environment by strategy interaction), it is strong causal evidence that nature influences digit span through the central executive, since another factor (strategy) that is also shown to influence digit span, interacts with nature in producing the digit span outcome.

### Study aims

We tested for a possible interaction between the effects of environmental exposure and cognitive strategy on executive functioning in three steps. In Experiment 1 we explicitly manipulated participants' cognitive strategies in a backward digit span task and a Raven's progressive matrices task in order to establish a link between cognitive strategy and executive mental function. The novelty of this experiment was twofold: (1) direct influences of instructional strategies on executive functioning have been claimed (Jacoby and Brooks, 1984; Smilek et al., 2006), but never directly tested, and (2) digit span performance was compared with a Raven's matrices task. Our hypothesis was that participants instructed to use an active strategy would show improved performance on backward digit and Raven's tasks, and that participants asked to use a passive strategy would show a decrement in performance. This experiment was therefore an essential step in establishing the conditions for our test of additive factors, since without it we would not know in Experiment 3 whether an instructional manipulation was effective on its own in influencing executive functioning.

In Experiment 2 we used a pair of 10-min video tours (urban, nature) to replicate previous findings between a relationship between environmental exposure and executive mental function (Berman et al., 2008, 2012). In this step we confirmed that our tasks were influenced in a similar way to past research by exposure to nature vs. urban environments. This experiment was also an essential step in establishing the conditions for our test of additive factors, since without it we would not know in Experiment 3 whether this environmental manipulation was effective on its own for influencing executive functioning. Also, in keeping with the current renewed emphasis on replicability and generalization in the behavioral sciences (Nosek et al., 2015), this experiment was important in establishing the robustness of the nature-executive functioning link.

In the critical third step, we combined these two manipulations in Experiment 3, to measure possible interactions between cognitive strategy and environmental exposure. Our hypothesis was that exposure to the nature video tour would reduce the influence of task instructions relative to the urban walking tour, thus supporting the hypothesis that the influence of nature and the influence of explicit strategies are being exerted on a common mental resource, namely central executive functioning.

## Experiment 1: strategy influences executive function

The purpose of this experiment was to assess the influence of explicit strategy instructions on two standard tasks used to index executive mental function, the backward digit span task and Raven's progressive matrices.

### Methods

#### Participants and design

The design of this experiment was a 2 (instruction: active, passive) × 2 (phase: pre, post) × 2 (task: BDS, Raven's) mixed experimental design, with instruction as the between-groups factor and phase and task as the within-groups factors. Ethical approval was granted by the University of British Columbia Behavioral Research Ethics Board for all studies in this paper (Certificate H14-00769) and all individuals gave informed consent in writing prior to participating in the study. All participants were recruited from the Department of Psychology human subject pool in return for extra course credit. In Experiment 1, 30 participants aged 18 to 30 years (23 female, 7 male, *M* = 21.80 years, *SD* = 2.88) were randomly assigned to active or passive conditions. The active and passive instructions were modified for the present tasks from those used in Smilek et al. (2006). Participants were told to either “actively direct your attention” or to use “your intuition,” and “gut-feeling” (see Appendix A.1, A.2 in Supplementary Material). The sample size of 30 was similar to previous studies on the role of instructions in altering task performance (Smilek et al., 2006).

#### Measures

Two executive function tasks were used in order (a) to generalize the findings and (b) to test for possible differences in the way executive functions are required for each task. A backward digit span task was adapted from the Wechsler Adult Intelligence Scale Third Edition (Wechsler, 1997). This task requires the use of working memory, a putative key component of executive function. The Raven's Progressive Matrices task uses images instead of learned symbols to test critical thinking and logic skills. Participants are shown a matrix of images with one cell left empty. They then attempt to choose the missing cell image from a selection of options displayed alongside the matrix. As the name implies, the task becomes more difficult with each additional matrix (Raven, 1989).

#### Stimuli

A backward digit span program was written for the Matrix Laboratory (MATLAB) software using the Psychophysics Toolbox (Brainard, 1997; Pelli, 1997; Kleiner et al., 2007). Participants were tested on 14 separate sequences of numbers, which started at three digits in length and increased by one digit every second trial up to our chosen maximum of nine digits. Each sequence was randomly generated and designed so that no sequence had duplicate digits. The digits were each shown in succession, and were each displayed, one at a time, and remained on the display for the length of 1 s. Between each digit a blank screen was displayed for 100 ms. Once the entire sequence had been displayed, the participant was presented with an on-screen box where they could input the reverse order of the digits shown. Each entirely correct sequence returned was scored as one point, and the maximum score a participant could achieve was 14. A second, more liberal scoring was also recorded, and counted one point per correct digit per sequence (e.g., if the shown sequence was 458 and the participant responded 438 then they would receive 2 points out of 3 when liberally scored, but would have received 0 out of 1 points when using the more conservative scoring). Conservative and liberal scoring systems were highly correlated, *r* = 0.934, *p* < 0.001, and as such only conservative scoring of our backward digit span task was used for comparison.

The Raven's task used in this experiment was written for MATLAB using the same Psychophysics Toolbox as the backward digit span task. The experiment used twenty-four 3 × 3 matrices, a subset of the entire available series. During an experimental pilot test we adjusted our subset selection of the matrices to attain a score of approximately 60% correct. The 24 selected matrices were then divided into odd and even numbered groups. Even numbered matrices were used in the pretest and odd numbered matrices were used in the posttest. Each matrix was displayed for a total of 60 s and the participant had to choose the missing tile from 8 options displayed on-screen concurrently with the corresponding matrix. Each correct trial was recorded as one point for a total possible score of 12 points in each pre- and posttest trials.

#### Procedure

Participants were tested on an Apple computer with a 27″ LCD monitor using a display resolution of 2560 × 1440. Participants were instructed on how to perform the backward digit span task and then given two practice trials to confirm that they understood the procedure. After performing the 14 backward digit span trials, the participants were shown a sample Raven's matrix and verbally instructed on how to perform the task. Once the participant understood the instructions, they were asked to complete 12 matrices, and to do so by answering each one within 60 s. Failing to respond in time resulted in the automatic displaying of the next matrix trial and the previous trial being scored as zero. Upon finishing the first two tasks, the participants were presented with the backward digit span instructions for their randomly assigned condition (active or passive). This was followed by a posttest backward digit span task, which was identical in design to the pretest, with 14 new trials. After the backward digit span was completed, the condition specific instructions for the Raven's task were shown. The participant then completed the posttest Raven's task, which was also identical in design to its pretest counterpart, with 12 different matrices shown.

## Results and discussion

Because the raw scores of the two tasks were based on different scales (i.e., a maximum score of 14 in the digit span task and 12 in the Raven's task), we used *z*-scores to compare performance across the two tasks and in combination. These *z*-scores were derived by subtracting the group mean for each task from the participant's raw score for that task, and then dividing this difference by the group standard deviation for the same task. Mixed design analyses of variance (ANOVA) were used to analyze the influences of the three factors (instruction, phase, task) on the *z*-scores. In addition, analyses of covariance (ANCOVA) were conducted, using pre-scores as a covariate, in order to control for potential baseline differences in performance between the two strategy conditions. These results only strengthened our conclusions in each experiment and so we report the ANOVA results in the body of the paper for simplicity and the ANCOVA results in the Appendix A.3 (Supplementary Material).

**Figure 2:**
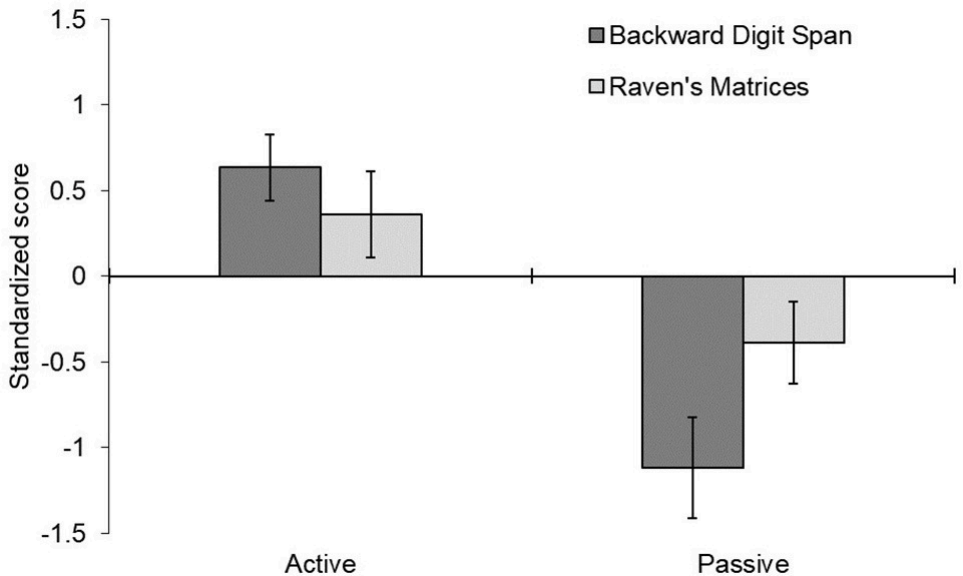
Mean difference in standardized scores before and after receiving cognitive strategy instructions. Error bars represent one standard error around the mean. Positive scores indicate improvement from pre- to post-testing; negative scores indicate a decrement over time.

The raw scores in the digit span and Raven task are shown in Table 1 and the pre-post difference scores are shown in Figure 2. Overall, the standardized scores for the digit span and the Raven's tasks were positively correlated, *r*(58) = 0.46, *p* < 0.001, supporting the hypothesis that there was significant overlap in the mental functions being tested in the two tasks.

**Table 1:** Mean scores in Experiment 1 by task and strategy instruction condition.

		**Backward digit**				**Raven's matrices**			
		**Pre**		**Post**		**Pre**		**Post**	
**Condition**	***n***	***M***	***SD***	***M***	***SD***	***M***	***SD***	***M***	***SD***
Active	15	6.93	2.84	9.13	2.07	5.60	2.17	6.47	2.30
Passive	15	7.67	3.48	3.80	3.12	5.40	2.38	4.47	2.56

*Increases from pre- to post-testing indicates a positive improvement in scores*.

The mixed-design ANOVA of the standardized scores indicated that the active instruction strategy led to generally better performance than the passive instruction strategy, *F*_(1, 28)_ = 5.480, *p* = 0.027, $\eta_{p}^{2}$ = 0.164. There was also a significant interaction of strategy x phase, *F*_(1, 28)_ = 20.508, *p* < 0.001, $\eta_{p}^{2}$ = 0.423, reflecting a general improvement from pre- to post-testing for the active strategy and a general reduction for the passive strategy. A three-way interaction of strategy x measure x phase, *F*_(1, 28)_ = 5.373, *p* = 0.028, $\eta_{p}^{2}$ = 0.161, reflected the finding that these strategy effects were stronger for the digit span task than the Raven's task. The digit span task showed a significant difference between active and passive conditions, *F*_(1, 28)_ = 24.45, *p* < 0.001, $\eta_{p}^{2}$ = 0.466, as did the Raven's task, *F*_(1, 28)_ = 4.205, *p* = 0.040, $\eta_{p}^{2}$ = 0.142, though the effect was larger in the digit span task, *F*_(1, 28)_ = 5.373, *p* = 0.028, $\eta_{p}^{2}$ = 0.161. All other effects were not significant, *p* > 0.30.

The main finding was that instructing participants to take an *active* approach led to an increase in performance, whereas instructing participants to take a *passive* approach reduced performance. The novel contribution of these findings is that this is the first time that passive-active instructions have been shown to directly influence backward digit span and Raven's Matrices, tasks that are tailored to make heavy demands on central executive processes. Specifically, active strategy instructions led to gains in performance, whereas passive strategy instructions led to performance decrements. A second novel contribution was finding that the influence of instructions were stronger for the backward digit span task than for Raven's matrices.

## Experiment 2: environment influences executive function

Here we repeated the pre-post design, but instead of inserting an instructional manipulation we invited participants to view a brief video of a nature tour through Banff National Park or an urban tour of the city of Barcelona.

### Methods

#### Participants and design

The design of this experiment was a 3 (video: natural, urban, none) × 2 (phase: pre, post) × 2 (task: BDS, Raven's) mixed experimental design, with video as the between-groups factor and phase and task as the within-groups factors. Ninety participants aged 17 to 45 years (68 female, 22 male, *M* = 21.10 years, *SD* = 3.54) were randomly assigned to either a nature video group, an urban video group, or a no video group. After completing Experiment 1, we used G^*^Power (Faul et al., 2007) to provide a post-hoc estimate of the statistical power of the main effect of strategy (Active vs. Passive) and the Strategy × Phase interaction (Pre-Post differences for Active vs. Passive). These estimates were power = 0.65 and 0.99, respectively. Based on these estimates, we doubled the number of participants in the three video conditions.

#### Stimuli and materials

A 10-min segment from a YouTube video of a Banff National Park tour was shown in the nature condition (www.youtube.com/watch?v=1Go2b40YsOw) and a 10-min segment of a Barcelona tour video was shown in the urban condition (https://www.youtube.com/watch?v=clW7aV0vVAY). These videos were selected because they each had numerous characteristics attributed to each of these two conditions in past research (e.g., reduced human impact in nature; artificial structures in urban) and because a video simulates the walking experience better than still photos. Both videos were shown in full screen mode, with the sound disabled. As with any comparison between environments, there were many differences to note. For example, there was a constant text banner in the lower left portion of the urban video but not in the nature video, consistent with the differences in text found in typical urban and natural settings. The videos were taken from different continents (North America, Europe) and the spacing and duration of camera cuts and pans clearly differed as well. Our goal was simply to see if these constellation of differences led to differences in executive functioning, as has been previously reported for different environments that vary on many of these dimensions as well. Finally, we note that in the no video condition participants moved directly from pre-testing to post-testing after a short break of 2–3 min, whereas each video took at least 10 min in the environment conditions (the basis of our critical comparison).

#### Procedure

Beyond the above differences, participants were tested using procedures identical to those used in Experiment 1.

## Results and discussion

**Figure 3:**
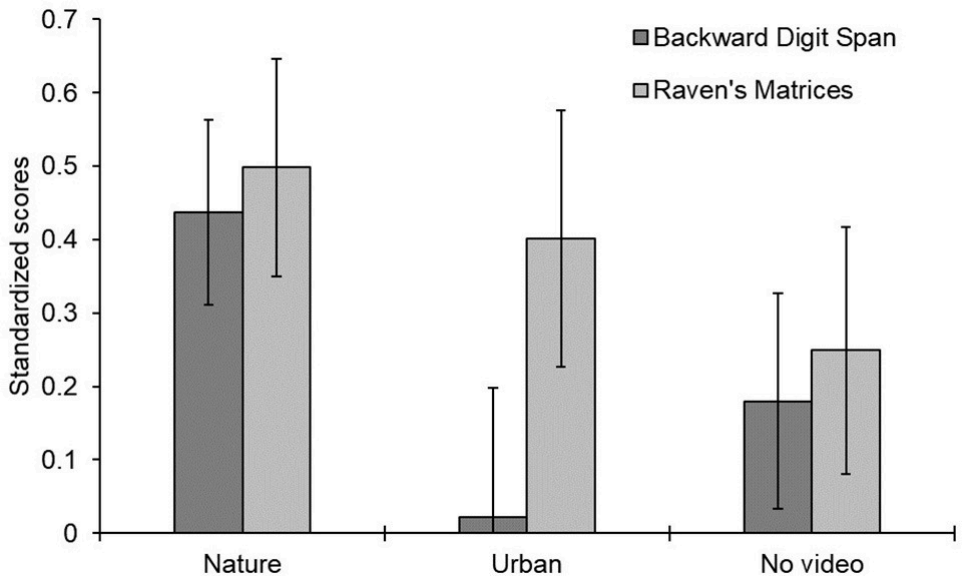
Mean difference in standardized scores before and after three different video viewing conditions. Error bars represent one standard error around the mean. Positive scores indicate improvement from pre- to post-testing; negative scores indicate a decrement over time.

The raw scores in the digit span and Raven task are shown in Table 2 and the pre-post difference scores are shown in Figure 3. A mixed-design ANOVA on the *z*-scores indicated that participants generally performed better on the posttest than on the pretest, *F*_(1, 87)_ = 21.728, *p* = 0.001, $\eta_{p}^{2}$ = 0.200, indicating a general improvement from pre- to post-testing for all **three** between-group conditions in this experiment.

**Table 2:** Mean scores in Experiment 2 by task and video viewing condition.

		**Backward digit**				**Raven's matrices**			
		**Pre**		**Post**		**Pre**		**Post**	
**Condition**	***n***	***M***	***SD***	***M***	***SD***	***M***	***SD***	***M***	***SD***
Active	30	7.53	2.78	8.83	2.52	4.47	2.06	5.67	1.92
Passive	30	7.73	3.17	7.80	3.31	5.27	2.26	6.23	2.94
No video	30	7.17	3.17	7.70	2.79	5.80	2.02	6.40	2.74

*Increases from pre- to post-testing indicates a positive improvement in scores*.

The two-way interaction of video x phase was not significant when all **three** video conditions were entered into the ANOVA model, *p* > 0.17, but it approached significance when the urban video and no video data were combined into a single group for comparison with the nature video condition, *F*_(1, 88)_ = 3.570, *p* = 0.062, $\eta_{p}^{2}$ = 0.039. This interaction supports the hypothesis that the nature video group improved more than the other groups from pre- to post-testing. As seen in Table 2, the nature video group showed the most consistent and largest improvement from pretest to posttest (all *p*-values Bonferroni corrected: digit span *t*_(29)_ = 3.47, *p* < 0.01; Ravens *t*_(29)_ = 3.36, *p* < 0.01), whereas the no video group showed the least improvement overall [digit span *t*_(29)_ = 1.22, *p* = 1.0; Raven's *t*_(29)_ = 1.48, *p* = 1.0], and the urban video group was mixed (i.e., no improvement in the digit span task, *t*_(29)_ = 0.128, *p* = 1.0, and some improvement in the Raven's task, *t*_(29)_ = 2.29, *p* = 0.06). When we examined each of the **two** tasks separately, the improvement in the digit span task following the nature video was significantly greater than for the urban and no video conditions, *F*_(1, 88)_ = 6.23, *p* = 0.01, $\eta_{p}^{2}$ = 0.066. The same comparison for the Raven's task was not significant, *F*_(1, 88)_ = 1.647, *p* > 0.30, $\eta_{p}^{2}$ = 0.018. All other effects were not significant, *p* > 0.30.

In summary, participants viewing the nature video improved in their task performance from pretest to posttest more than participants who viewed either an urban video or no video. This finding is consistent with previous tests of attention restoration theory (Berto, 2005; Berman et al., 2008; Berto et al., 2010). The novel finding is that there was greater support for the theory in the backward digit span than in the Raven's Matrices task. This may suggest that the digit span task makes heavier demands on executive functioning than the Raven's task.

## Experiment 3: strategy and environment interact in their influence

The experiment tested environmental exposure via brief video tours in combination with strategy instructions. The goal was to determine whether the effects seen in Experiments 1 and 2 influenced the same or different cognitive mechanisms, using additive factors logic (Sternberg, 1969, 1998, 2001; Ghorashi et al., 2009). Sternberg proposed that separate factors interact when they operate on a common underlying mechanism and act additively when they operate on separate mechanisms. Following the logic illustrated in Figure 1, we hypothesized that if both factors (i.e., strategy and environment) influenced the same latent construct, then the factors should interact in their effects on task performance. That means that in addition to any main effects of strategy and environment, we should also see an interaction, such that a nature video should reduce the difference in strategy effects relative to the urban video. Conversely if the underlying mechanisms were unique, then additive results would be predicted. Only the main effects should be present, consistent with each factor having independent (unrelated) effects on executive functioning. The specific interaction predicted by attention restoration theory (Kaplan, 1995; Berman et al., 2008; Kaplan and Berman, 2010), is that the nature video, in comparison to the urban video, should reduce the difference between the two instructional strategies. This is because interacting with nature is believed to restore central processing resources, leaving them more fully available for use under both kinds of instruction. In contrast, interacting with an urban environment should deplete these resources, and increase the gap between participants encouraged to take a passive vs. an active approach to the tasks.

### Methods

#### Participants and design

The design of this experiment was a 2 (video: natural, urban,) × 2 (instruction: active, passive) × 2 (phase: pre, post) × 2 (task: BDS, Raven's) mixed experimental design, with video and instruction as between-groups factors and phase and task as the within-groups factors. Eighty participants aged 18–63 years (56 female, 24 male, *M* = 23.18 years, *SD* = 8.05) were given the tasks, instructions, and videos used in the previous experiments. Half the participants in each video condition were randomly assigned to receive the active instructions, and the rest were assigned to receive the passive instructions. Because we were looking for a possible three-way interaction between strategy x video x phase in this experiment, we again increased the sample in each of the two instructional conditions to *n* = 40.

#### Stimuli and procedure

When all participants had been pre-tested in the two tasks, they were either shown the urban or the nature video tour. Following the completion of the video, the strategy instructions were administered in anticipation of the post-test.

## Results and discussion

**Figure 4:**
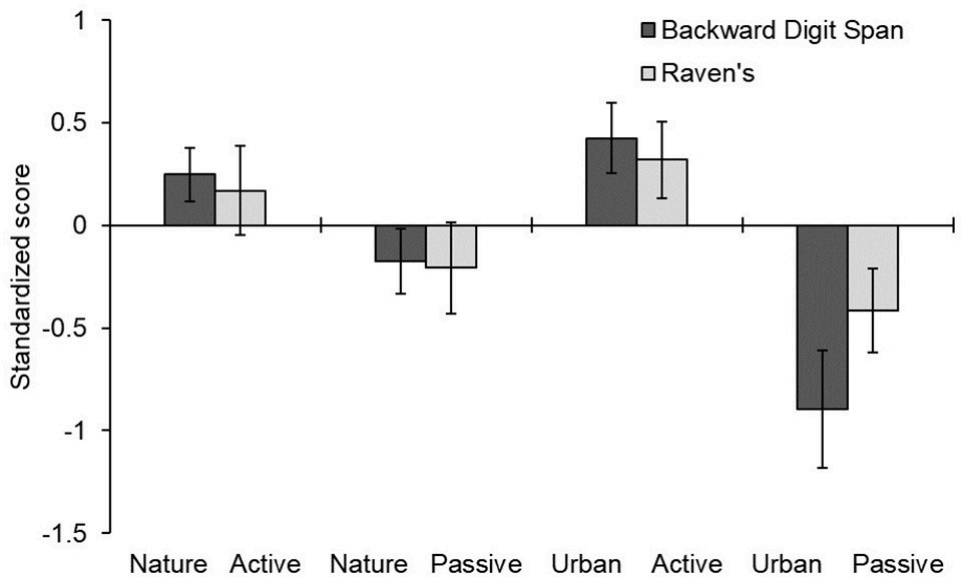
Mean difference in standardized scores before and after four combinations of strategy and video viewing conditions. Error bars represent one standard error around the mean. Positive scores indicate improvement from pre- to post-testing; negative scores indicate a decrement over time.

The raw scores in the digit span and Raven task are shown in Table 3 and the pre-post difference scores are shown in Figure 4. A mixed-design ANOVA on the *z*-scores indicated no main effects of video, instruction, or phase (all *p*-values > 0.25). However, there was a significant interaction of strategy x phase, *F*_(1, 76)_ = 22.903, *p* = 0.001, $\eta_{p}^{2}$ = 0.232, reflecting that participants generally improved from pretest to posttest when given the active strategy instructions [Bonferroni corrected *t*_(76)_ = 2.57, *p* < 0.01] and they generally declined from pretest to posttest when given the passive instructions [*t*_(76)_ = 2.80, *p* < 0.01). This replicates the main finding of Experiment 1. However, more important for our central hypothesis, was the significant three-way interaction of strategy x video x phase, *F*_(1, 76)_ = 4.407, *p* = 0.039, $\eta_{p}^{2}$ = 0.055. As shown in Figure 4, this interaction indicated that the difference between active and passive strategies was larger in the urban video condition [Bonferroni corrected *t*_(76)_ = 4.87, *p* < 0.01) than in the nature video condition [*t*_(76)_ = 1.90, *p* = 0.37]. These results imply that viewing a nature video plays a buffering role against cognitive strategies, modulating both the strong positive (for active instructions) and negative (for passive instructions) influences of adopting a particular cognitive strategy. No effects involving measure were significant in this experiment, *p* > 0.24.

**Table 3:** Mean scores in Experiment 3 by task, video, and strategy condition.

		**Backward digit**				**Raven's matrices**			
		**Pre**		**Post**		**Pre**		**Post**	
**Condition**	***n***	***M***	***SD***	***M***	***SD***	***M***	***SD***	***M***	***SD***
Active nature	20	6.25	3.43	7.10	3.82	5.20	2.33	5.65	3.39
Active urban	20	7.50	2.88	8.95	2.63	6.90	2.36	7.75	2.15
Passive nature	20	6.55	2.96	5.95	3.09	5.55	2.26	5.00	2.22
Passive urban	20	8.00	3.35	5.00	3.81	6.00	2.53	5.00	2.92

*Increases from pre- to post-testing indicates a positive improvement in scores*.

## General discussion

The main aim of this study was to test a central tenet of attention restoration theory, namely, that exposure to a natural vs. urban environment has a direct positive influence on executive mental function (also known as working-memory and directed attention, Kaplan and Talbot, 1983; Kaplan, 1995; Kaplan and Berman, 2010). Previously, this link had been drawn on the grounds that tasks designed to index executive function (e.g., backward digit span) were influenced in a predictable way by exposure to nature vs. urban settings. But a direct influence on executive functioning is not the only way that exposure to nature could lead to improved performance on tasks such as digit span, as illustrated in Figure 1. For example, it could come about through a change in sensory registration processes that occur prior to central executive processes, or in decision processes that occur after these processes have occurred, or in a host of other potential latent variables such as a change in general motivation or effort applied to the task.

In the present study we used the manipulation of cognitive strategy, implemented through the administration of task instructions, which have previously been argued to influence the degree to which executive control is used to perform tasks of categorization (Jacoby and Brooks, 1984; Whittlesea et al., 1994), visual search (Smilek et al., 2006; Watson et al., 2010), and rapid serial target detection (Olivers and Nieuwenhuis, 2005). The novel contribution of Experiment 1 was to show that passive-active instructions had the predicted influence on two tasks that are tailored to make heavy demands on central executive processes (i.e., backward digit span and Raven's Matrices).

The second experiment was a conceptual replication of the main finding of previous tests of the attention restoration theory, with an extension to a brief video tour instead of photos or a walk (Berto, 2005; Berman et al., 2008, 2012), and a comparison of the backward digit span task with Raven's Matrices. The results supported the hypothesis in general, but showed stronger environmental exposure effects for digit span task than the Raven's task. We suspect this is because the digit span tasks places even greater demands on conscious control processes than Raven's Matrices. Because the Raven's task involves visual patterns, there is the opportunity for unconscious and spatially parallel processes of visual pattern matching to contribute to performance (Soulières et al., 2009). The digit span task, on the other hand, relies entirely on operations within working memory (mental space), since the digits are no longer visible on the screen when the correct answer must be reconstructed from memory.

These two findings placed us in a position to perform the main novel test of this study, which was to see whether instructional effects were interactive or additive with the environmental effects. The nature video tour attenuated (reduced) the influence of task instructions relative to the urban video tour. As such, these findings provide strong support for one of the main tenets of attention restoration theory, namely, that brief exposure to nature influences executive mental functions directly rather than via another motivational or strategic route.

### Implications for attentional restoration theory

While this study does not rule out other potential mechanisms that very well could also be at play, such as stress reduction, motivation, and perceptual fluency, the results support the view that at least some of the positive cognitive effects derived from viewing nature involved changes to the executive control of attention. As such, these data provide compelling support for attention restoration theory and its hypothesized mechanism for the cognitive benefits of nature, which are influences on the executive control of attention (i.e., directed attention).

### Limitations

This study is only a first step in applying additive factors logic to the question of how exposure to nature exerts an influence on human cognition. Many other potential factors and latent variables remain to be explored, following the logic outlined in Figure 1. For example, it will be important in the future to test tasks of cognition that are *not* theoretically linked to central executive functioning. One example might be a pop-out visual search, such as was tested in Smilek et al. (2006). If exposure to nature influences central executive functioning and a pop-out search task does not require those functions, then one would expect no interaction between environmental exposure (nature, urban) and the level of difficulty in pop-out search tasks (faster, slower).

Critics might also question some of the assumptions underlying the design of this study. For example, is it really the case that exposure to a natural environment has an *exogenous* influence on central executive task performance? Might the effects of nature also be intentional, volitional, and therefore provide a better fit to the definition of *endogenous* control of attention? If so, then it would be less of a surprise that environments interacted with strategies in their influence, since both would be endogenous effects. We think the answer to this question is a strong “no,” because the conventional definition of endogenous is that it involves volitional intent (meaning a choice that the participants are consciously aware of) and controlled processing (meaning with conscious effort). The prevailing view in the literature on environmental effects is that they occur outside of the awareness of participants (Berman et al., 2008, 2012; Kaplan and Berman, 2010). At the same time, it would be prudent to test this assumption more directly in future studies. This could be done by either administering a structured interview to participants following an environmental manipulation, or by probing participants with surprise questions during the performance of the executive functioning tasks.

Another potential criticism of the study concerns the differences between the cognitive strategies employed by participants. It might be argued that the passive instructions encourage participants to simply apply less effort to the task than the active instructions, such that the strategy manipulation is one of motivation or effort rather than of cognitive style. We have two responses to this criticism. First, ample past research has shown that passive instructions lead to better performance than active performance in some difficult and speeded cognitive tasks (Olivers and Nieuwenhuis, 2005; Smilek et al., 2006; Watson et al., 2010). If this was simply a manipulation of motivation or effort then one would expect active instructions to always lead to better performance. Instead, the conclusion consistent with all past research using strategy manipulations of this kind, including the present study, is that active instructions encourage greater reliance on central executive processes than passive instructions.

A second response is that the effort interpretation of strategy effects is inconsistent with the interaction observed in this study between environmental exposure and strategy. Reduced effort in the passive condition should have resulted in an additive pattern of results, with environmental exposure showing its influence independent of the effects of effort or motivation. Each of these effects should have simply added together. Instead, the present findings demonstrate that environmental exposure and strategy modulate each other's influence, consistent with them each drawing on a common pool of cognitive resources. In the present study that meant that the consequences of strategy instructions were reduced in the nature video condition compared to the urban video, consistent with nature ameliorating the otherwise detrimental effects of adopting a passive instructional strategy when confronted with difficult tasks requiring executive control.

## Conclusion

Previous studies have provided considerable evidence for the positive influence of exposure to natural environments on central executive functions such as working memory and response inhibition (Berman et al., 2008, 2012; Kaplan and Berman, 2010). However, this evidence has been largely based on the finding that time spent in or viewing nature has positive effects on tasks thought to require executive functioning for their successful completion. In this study we sought and found stronger evidence for this link based on additive factors logic. Using this approach, we first of all established that two experimental factors (strategy instructions in Experiment 1 and environmental exposure in Experiment 2) each independently influenced performance on two executive function tasks (backward digit span memory and Raven's matrices). Then, in Experiment 3, these two manipulations were combined with the main finding that viewing a brief nature video tour attenuated the influence of task instructions relative to an urban video tour. This study therefore adds important evidence to the main claim of attention restoration theory: that exposure to nature has a direct and positive influence on executive mental functioning. Challenges for future studies will include focusing on the specific components of central executive function (e.g., working memory capacity, operational capacity, inhibition, cognitive flexibility) that are influenced in this way and understanding how this influence comes about (e.g., mediation by positive emotion, stress reduction, recovery from mental fatigue).

## Ethics statement

This study was carried out in accordance with the recommendations of The University of British Columbia Behavioral Research Ethics Board with written informed consent from all subjects. All subjects gave written informed consent in accordance with the Declaration of Helsinki. The protocol was approved by The University of British Columbia Behavioral Research Ethics Board.

## Author contributions

This research formed part of the MA thesis of SB at the University of British Columbia (2015). JE and MB assisted in the theoretical question, experimental design of the study, and in the writing of this paper. Otherwise all data collection, data analysis, and writing were overseen by SB. This research was supported by a Discovery Grant to JE, from the Natural Sciences and Engineering Research Council of Canada.

### Conflict of interest statement

The authors declare that the research was conducted in the absence of any commercial or financial relationships that could be construed as a potential conflict of interest.
