# Supplementary material for: Cognitive Strategies and Natural Environments Interact in Influencing Executive Function
Source: Front Psychol. 2018 Jul 23;9:1248. doi: 10.3389/fpsyg.2018.01248 (PMC6064875; doi:10.3389/fpsyg.2018.01248)
Supplement: Supplementary file 1 [file Data_Sheet_1.pdf]

APPENDIX

A.1 Active Instructions

Backward digit span

The best strategy for this task, and the one that we want you to use in this study, is to try to actively direct your attention to each number you are shown and asked to recall. The idea is to actively direct your attention to each number. Sometimes people find it difficult or strange to "direct their attention" but we would like you to try your best. Try to respond as quickly and accurately as you can while using this strategy. Remember, it is critical for this experiment that you actively direct your attention to each number you are shown and asked to remember.

Raven's progressive matrices

The best strategy for this task, and the one that we want you to use in this study, is to work at actively directing your attention to the patterns. The idea is to use this strategy to direct your attention to each set of pattern in order to find the missing one. Sometimes people find it difficult or strange to "direct their attention" but we would like you to try your best. Try to respond as quickly and accurately as you can while using this strategy. Remember, it is very critical for this experiment that you actively direct your attention in order to determine the missing pattern.

A.2 Passive Instructions

Backward digit span

The best strategy for this task, and the one that we want you to use in this study, is to relax your attention in order to let the numbers you are shown and asked to recall just "pop" into your mind. The idea is to let your intuition determine your response. Sometimes people find it difficult or strange to tune into their "gut feelings" but we would like you to try your best. Try to respond as quickly and accurately as you can while using this strategy. Remember, it is very critical for this experiment that you relax so that the numbers to recall just "pop" into your mind.

Raven's progressive matrices

The best strategy for this task, and the one that we want you to use in this study, is to relax your attention in order to let the missing pattern just "pop" into your mind. The idea is to let your intuition determine your response. Sometimes people find it difficult or strange to tune into their "gut feelings" but we would like you to try your best. Try to respond as quickly and accurately as you can while using this strategy. Remember, it is very critical for this experiment that you relax so that the missing pattern just "pops" into your mind.

A.3 Analyses of covariance

Experiment 1. We conducted analyses of covariance (ANCOVA), using pre-scores as a covariate, in order to control for potential baseline differences in performance between the two strategy conditions. The results only strengthened our conclusions. The digit span task showed a significant difference between active and passive conditions, $F(1, 27) = 35.50$, $p < .001$, $\eta_p^2 = .568$, after accounting for significant differences in pretest scores, $F(1, 27) = 22.39$, $p < .001$, $\eta_p^2 = .453$. Active instructions also led to improved accuracy in the Raven's task, $F(1, 28) = 6.04$, $p = .021$, $\eta_p^2 = .183$, after accounting for significant differences in pretest scores, $F(1, 27) = 6.233$, $p < .019$, $\eta_p^2 = .188$. We also examined the post-test scores directly, after adding pre-scores as a covariate, which is an equivalent way to examine change scores in the context of baseline differences in pre-scores (Jamieson, 2004). These analyses led to the same conclusions as those reported here, both in this experiment and in the ones that follow.

Experiment 2. We conducted ANCOVA as in Experiment 2. The results again strengthened our conclusions. The digit span task showed a significant difference between urban and nature conditions, $F(1, 87) = 4.49$, $p < .037$, $\eta_p^2 = .049$, after accounting for significant difference in pretest scores, $F(1, 87) = 24.34$, $p < .001$, $\eta_p^2 = .219$. The difference between video conditions was not significant in the Raven's task, $F(1, 87) < 1$, $p = .825$, $\eta_p^2 = .001$, after accounting for significant difference in pretest scores, $F(1, 87) = 7.71$, $p < .01$, $\eta_p^2 = .081$.

Experiment 3. We conducted ANCOVAs, after adding pre-scores as a covariate, in order to control for baseline differences in performance between the two strategy conditions. The results again supported our conclusions. The interaction of environment x strategy was significant in the digit span task, $F(1, 75) = 5.47$, $p = .022$, $\eta_p^2 = .068$, after accounting for significant difference in pretest scores, $F(1, 75) = 14.18$, $p < .001$, $\eta_p^2 = .159$. This interaction was not significant in the Raven's task, $F(1, 75) = 1.79$, $p = .185$, $\eta_p^2 = .023$, after accounting for significant difference in pretest scores, $F(1, 75) = 11.99$, $p < .001$, $\eta_p^2 = .138$.
